# Rapid Detection of *DNMT3A* R882 Mutations in Hematologic Malignancies Using a Novel Bead-Based Suspension Assay with BNA(NC) Probes

**DOI:** 10.1371/journal.pone.0099769

**Published:** 2014-06-10

**Authors:** Velizar Shivarov, Milena Ivanova, Elissaveta Naumova

**Affiliations:** 1 Laboratory of Hematopathology and Immunology, National Hematology Hospital, Sofia, Bulgaria; 2 Department of Clinical Immunology, Alexandrovska University Hospital, Medical University, Sofia, Bulgaria; Queen's University Belfast, United Kingdom

## Abstract

Mutations in the human DNA methyl transferase 3A (*DNMT3A*) gene are recurrently identified in several hematologic malignancies such as Philadelphia chromosome-negative myeloproliferative neoplasms (MPN), myelodysplastic syndromes (MDS), MPN/MDS overlap syndromes and acute myeloid leukemia (AML). They have been shown to confer worse prognosis in some of these entities. Notably, about 2/3 of these mutations are missense mutations in codon R882 of the gene. We aimed at the development and validation of a novel easily applicable in routine practice method for quantitative detection of the *DNMT3A* p.R882C/H/R/S mutations bead-based suspension assay. Initial testing on plasmid constructs showed excellent performance of BNA(NC)-modified probes with an optimal hybridization temperature of 66°C. The method appeared to be quantitative and showed sensitivity of 2.5% for different mutant alleles, making it significantly superior to direct sequencing. The assay was further validated on plasmid standards at different ratios between wild type and mutant alleles and on clinical samples from 120 patients with known or suspected myeloid malignancies. This is the first report on the quantitative detection of *DNMT3A* R882 mutations using bead-based suspension assay with BNA(NC)-modified probes. Our data showed that it could be successfully implemented in the diagnostic work-up for patients with myeloid malignancies, as it is rapid, easy and reliable in terms of specificity and sensitivity.

## Introduction

Somatic mutations in the DNA methyltransferase 3A (DNMT3A) gene have been reported as recurrently associated with myeloid malignancies. The frequency of mutations varies between different entities. In adult acute myeloid leukemia (AML) *DNMT3A* mutations are found in 14–34% of cases from different series [1], 5–15% of MDS cases [2], 10% of chronic myelomonocytic leukemia (CMML) patients [3], 5.7% of primary myelofibrosis (PMF) patients [4], 12% of cases with systemic mastocytosis [5] and about 18% of T cell acute lymphoblastic leukemia (T-ALL) cases [6]. Approximately 2/3 of the *DNMT3A* are missense mutations at codon R882 [7]. Most of the current literature supports the notion that the presence of *DNMT3A* mutations is an adverse prognosis biomarker at least in adult AML and may be an important parameter of the integral molecular genetics profiling in these patients [8], [9], [10]. These lines of evidence necessitate the development of methods for reliable detection of *DNMT3A* mutations that are easily applicable and affordable for routine use together with the testing for other mutations. The most frequently reported techniques used for *DNMT3A* mutations include direct sequencing, high-resolution melting analysis and next generation sequencing. Sanger sequencing is particularly well-established technique [7], [11] for the identification of previously unreported mutations but its relatively low sensitivity (∼10–20% mutant allele burden) may be problematic for detection of low frequency somatic mutations. High resolution melting (HRM) technique has also been adapted for detection of *DNMT3A* mutations and is also good for screening for unknown mutations in a single tube format with a sensitivity of about 4% [12], [13]. However, it requires well-established standards and eventually verification of the result through direct sequencing. Targeted amplicon resequencing on next generation sequencing (NGS) platforms has also been used for *DNMT3A* mutations [14], [15]. The obvious advantages of NGS as the digital allele burden output, theoretically very high sensitivity, the possibility for identification of novel mutations are currently limited by the costly equipment and the necessity for a strong bioinformatic support. Simple approaches such as restriction fragment length polymorphism (RFLP) based assay have also been developed [16], whose relatively low sensitivity could be overcome by coupling with quantitative PCR (qPCR)[17]. Another less frequently used technique for verification of *DNMT3A* mutations confirmation was denaturing high-performance liquid chromatography (DHPLC) [18].

In our view a particularly suitable for routine diagnostics could be the detection of somatic mutations in hematologic malignancies using a microsphere-based suspension array. It allows single tube quantitative detection of mutations in one or several amplicons in a mid-throughput format, making it a rapid and affordable method. Here we report the development and validation of a sensitive multiplexed bead-based suspension assay for quantitative detection of *DNMT3A* R882 mutations. The method appeared to be applicable to clinical samples from patients with myeloid malignancies.

## Materials and Methods

### Ethics statement

The study was conducted in accordance with the principles of the Declaration of Helsinki. Written informed consent was obtained from all patients. Blood and bone marrow sampling as well as molecular testing were part of the routine diagnostic procedures approved by the Institutional Review Board (IRB) at Alexandrovska University Hospital, Sofia, Bulgaria.

### Patients

A total of 120 peripheral blood or bone marrow samples of consecutive patients with known or suspected myeloid malignancies were collected between January 2010 and June 2013. Patients were classified according to the WHO criteria as follows: Acute myeloid leukemia (AML) (n = 21), Chronic myeloid leukemia (CML) (n = 1), Myelodysplastic syndrome (MDS) (n = 6), Myeloproliferative neoplasm (MPN) (n = 81), Overlap MPN/MDS (n = 5), and others with suspected but unproven MPN (n = 6).

### DNA extraction

All samples were collected using sodium citrate-containing blood sampling tubes (BD Biosciences) and stored at room temperature for no more than 4 hours before processing. Genomic DNA was extracted from whole blood using a iPrep (Invitrogen) automated system. Genomic DNA samples were stored at −20°C before further analyses.

### *DNMT3A* exon 23 sequencing

Exon 23 of the human *DNMT3A* gene was amplified from genomic DNA as reported before [5] using the following primers: DNMT3A (forward): 5′-TCCTGCTGTGTGGTTAGACG and DNMT3A (reverse): 5′-ACAGAAAACCCCTCTGAAAAG. Amplification in 25 µl reactions included the following: 100 ng genomic DNA; 1.5 U Taq polymerase (Invitrogen); 3 mM MgCl_2_; 0.2 mM dNTP mixture; and 10 pmol of each primer. The amplification conditions were as follows: 95°C for 5 min; 35 cycles of 95°C for 30 sec, 58°C for 40 sec, 72°C for 2 min and final extension at 72°C for 10 min. PCR products were purified using Exo-SAP (Applied Biosystems, USA) and sequenced bidirectionally with the same set of primers. The sequencing reaction was performed in a final volume of 10 µL using 2 µL of the purified PCR product, 3.2 pmol of one the primers and 2 µL of Big Dye terminator cycle-sequencing kit v3.1 (Applied Biosystems, USA). The sequencing program was 25 cycles of denaturation at 96°C for 10 s, annealing at 50°C for 5 s and extension at 60°C for 4 min. The sequence detection was conducted using the ABI Prism 3100 Genetic Analyzer (Applied Biosystems, USA). Sequences alignment and analysis were performed using Sequencher v. 5.0. (GeneCodes, USA).

### Bead-based assay

The bead-based assay was performed as described before [19], [20], [21]. Briefly, the exon 23 *DNMT3A* fragment was amplified from either genomic or plasmid DNA samples using a 5′-botinylated forward primer. The same primers and PCR conditions as described above for the sequencing analysis were applied. Genotyping was performed by direct hybridization with 5 LNA- or BNA(NC)-modified oligonucleotide probes, specific for the wild type or the mutant alleles. LNA-modified oligonucleotides were designed and synthesized by Exiqon, Denmark, and the BNA(NC)-modified oligonucleotides were designed by us and synthesized by BioSynthesis, USA. The sequences and the melting temperatures (Tm) of all oligonucleotides are shown in Table 1. All probes were synthesized with 5′-amino group and 20 bases as a spacer sequence for the purpose of covalent binding to the carboxylated microspheres (Luminex, USA). The coupling of the amine modified probe to the carboxylated surface of the beads was performed using a standard carbodiimine-coupling procedure [22]. Mixtures of 5 sets of coupled microspheres were prepared by combining equal volumes of each set of the microspheres. Approximately, 160 beads of each type/µl were used for the analysis of one sample. Five µl of PCR product; 20 µl Hybridization buffer (Wakunaga Pharmaceuticals, Japan); 3 µl Bead mixture and 2 µl of streptavidin-phycoerythrine (SAPE) (Wakunaga Pharmaceuticals, Japan) were combined per well of a 96-well Thermowell plate and hybridization was performed at 62°C, 64°C, 66°C or 68°C for 30 min in a thermocycler (Applied Biosystems, USA). After incubation, 75 µl Washing buffer (Wakunaga Pharmaceuticals, Japan) were added to each well. The supernatants were removed after plate centrifugation at 4000 rpm for 2 min. Finally, the microspheres were resuspended in 75 µl Washing buffer (Wakunaga Pharmaceuticals, Japan) and acquired on a LabScan^200^ flow platform (Luminex, USA). A minimum of 100 events per bead region of interest was collected. For each set of reactions a background control of a sample containing only the respective sets of microspheres was included. The background median fluorescence intesity (MFI) values were subtracted from the MFI values for each sample. The resulting values were used for the calculation of the relative fluorescence indices for each mutant allele according to the following equation: Index (mutant allele*_i_*)  =  MFI (mutant allele*_i_*)/[MFI (mutant allele*_1_*) + … +MFI (mutant allele*_4_*) + MFI (wild type allele)].

**Table 1:** Primary sequence of the tested oligonucleotides and the predicted melting temperatures (Tm).

Modification	Probe designation/Codon change	Sequence	Tm
LNA	WT (CGC)	5′-CGCCAAGCGGCTCAT-3′	68°C
	R882C (CGC->TGC)	5′-CGCCAAGCAGCTCAT-3′	66°C
	R882H (CGC->CAC)	5′-CGCCAAGTGGCTCAT-3′	66°C
	R882P (CGC->CCC)	5′-CGCCAAGGGGCTCAT-3′	67°C
	R882S (CGC->AGC)	5′-CGCCAAGCTGCTCAT-3′	66°C
BNA(NC)	WT (CGC)	5′-CGCCA+AGCG+GCTCATGTT-3′	70°C
	R882C (CGC->TGC)	5′-CGCC+A+AGCAG+CTC+ATGTT-3′	70°C
	R882H (CGC->CAC)	5′-CGCC+A+AGTG+GCTC+ATGTT-3′	70°C
	R882P (CGC->CCC)	5′-CGCCA+AGGGG+CTCATGTT-3′	70°C
	R882S (CGC->AGC)	5′-CGCC+A+AGCTG+CTC+ATGTT-3′	70°C

The character “+N” denotes the position of any BNA(NC) modified nucleotide. The positions of LNA modification are proprietary property of Exiqon, Denmark and are, therefore, not disclosed.

### Determination of the cut-off index values of the assay

Sensitivity and specificity of the assay for each mutant as well as the optimal threshold values for mutant allele indices were determined using Receiver Operating Characteristic (ROC) analysis [23]. All indices for positive and negative samples for each mutant from five independent runs of the assay were used for the analysis. The optimal cut-off point was determined by the Fisher's test implemented through the R language-based web tool Cutoff Finder (http://molpath.charite.de/cutoff/) [24]. Analysis of the sensitivity in terms of percent mutant allele burden was performed by plotting the range of indices for each mutant at concentration 1%, 2.5% and 5% and 12.5% versus the already determined optimal cut-off value.

## Results

### Determination of the optimal probes set and hybridization conditions

The development of any hybridization-based assay for detection of single nucleotide changes (e.g. SNPs, point mutations) is particularly challenging because of the need to design highly specific probes that are able to discriminate between the different alleles with high accuracy. The task becomes even more complicated when more than two alleles are to be discriminated in a multiplex fashion. The design of the probes is also influenced by the overall nucleotide content of the fragment of interest and in some instances even single nucleotide changes can lead to a high variability in the melting temperatures. So, the primary goal of the optimal probe set design is to select probes with high and uniform Tm. Traditionally, this could be achieved through variation of probe length, which is a time and labor-consuming process. To overcome this limitation a number of chemically modified bases have been used to design oligonucleotide probes. For the purpose of developing a bead-based assay for detection of *DNMT3A* R882 mutations we tested the performance of two sets of oligonucleotide probes with synthetically modified nucleotides. The first set comprised of 1^st^ generation bridged nucleic acids (BNA), known also as locked nucleic acids (LNA)-oligonucleotides, *i.e.* they contain one or more 2′-O,4′-C-methylene-β-D-ribofuranosyl monomer(s) [25]. These probes were designed and synthesized by the LNA patent holder Exiqon, Denmark. The second set of in-house designed probes included third generation BNA oligonucleotides, known also as 3^rd^ generation bridged nucleic acids (BNA(NC)), *i.e.* they contain more than one 2′-O,4′-aminoethylene-β-D-ribofuranosyl monomers [26]. The design of the BNA(NC) oligonucleotides was performed following the published recommendations (http://www.biosyn.com/faq/BNA-general-design-guidelines.aspx) and they were synthesized by BioSynthesis Inc., USA.

**Figure 1:**
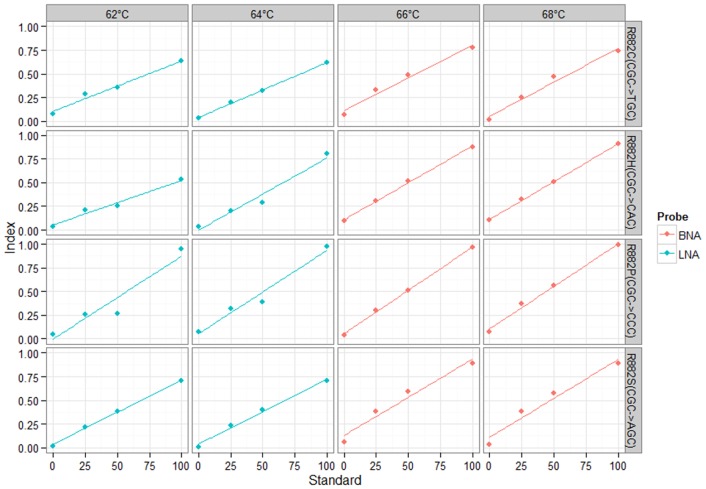
Fitted linear standard curves from the test assays for determination of the best performing probe set and the optimal hybridization temperature.

As shown in Table 1 the BNA(NC) probes had uniform Tm of 70°C, whereas the LNA probes had slightly lower Tm values varying between 66°C and 68°C. Based on our previous experience with Luminex-based hybridization assays[21] we expected the optimal performance of each set of probes to be around 2°C–4°C below the expected Tm. Indeed, to determine the optimal hybridization conditions for each set of probes we used dilutions of 100%, 50%, 20%, and 0% of each mutant plasmid in the wild-type plasmid. For the LNA probes set we tested 62°C and 64°C hybridization temperatures and for the BNA(NC) probes we tested 66°C and 68°C hybridization temperatures. Mutant allele indices for each sample were calculated and linear regression model was fitted for each mutant at every hybridization temperature (Fig. 1). The parameters for every linear model fit are presented at Table 2. The BNA(NC) probes set performed better than the LNA probes and the former was selected for further testing. The 66°C and 68°C hybridization temperature levels tested for the BNA(NC) probes were actually identical in terms of the parameters of the fitted linear models (Table 2). We selected the 66°C for all subsequent runs of the assay as at this level the raw MFI values were higher than those at the 68°C level.

**Table 2:** Parameters of the fitted linear models shown in Fig. 1.

Modification	LNA							
Temperature	62°C				64°C			
Mutant	R882C	R882H	R882P	R882S	R882C	R882H	R882P	R882S
Intercept	0.1041	0.0526	−0.0033	0.0322	0.0393	−0.0017	0.0535	0.0373
Slope	0.0053	0.0047	0.0088	0.0068	0.0058	0.0076	0.0088	0.0069
R^2^	0.9812	0.9717	0.9179	0.9972	0.9984	0.966	0.967	0.9922

Modification	BNA(NC)							
Temperature	66°C				68°C			
Mutant	R882C	R882H	R882P	R882S	R882C	R882H	R882P	R882S
Intercept	0.1139	0.1074	0.0525	0.1277	0.0508	0.1105	0.1045	0.1086
Slope	0.0069	0.0078	0.0092	0.0081	0.0072	0.008	0.009	0.0082
R^2^	0.9779	0.9976	0.999	0.9648	0.9793	0.9996	0.9932	0.964

### Determination of the index threshold value and assay sensitivity

**Figure 2:**
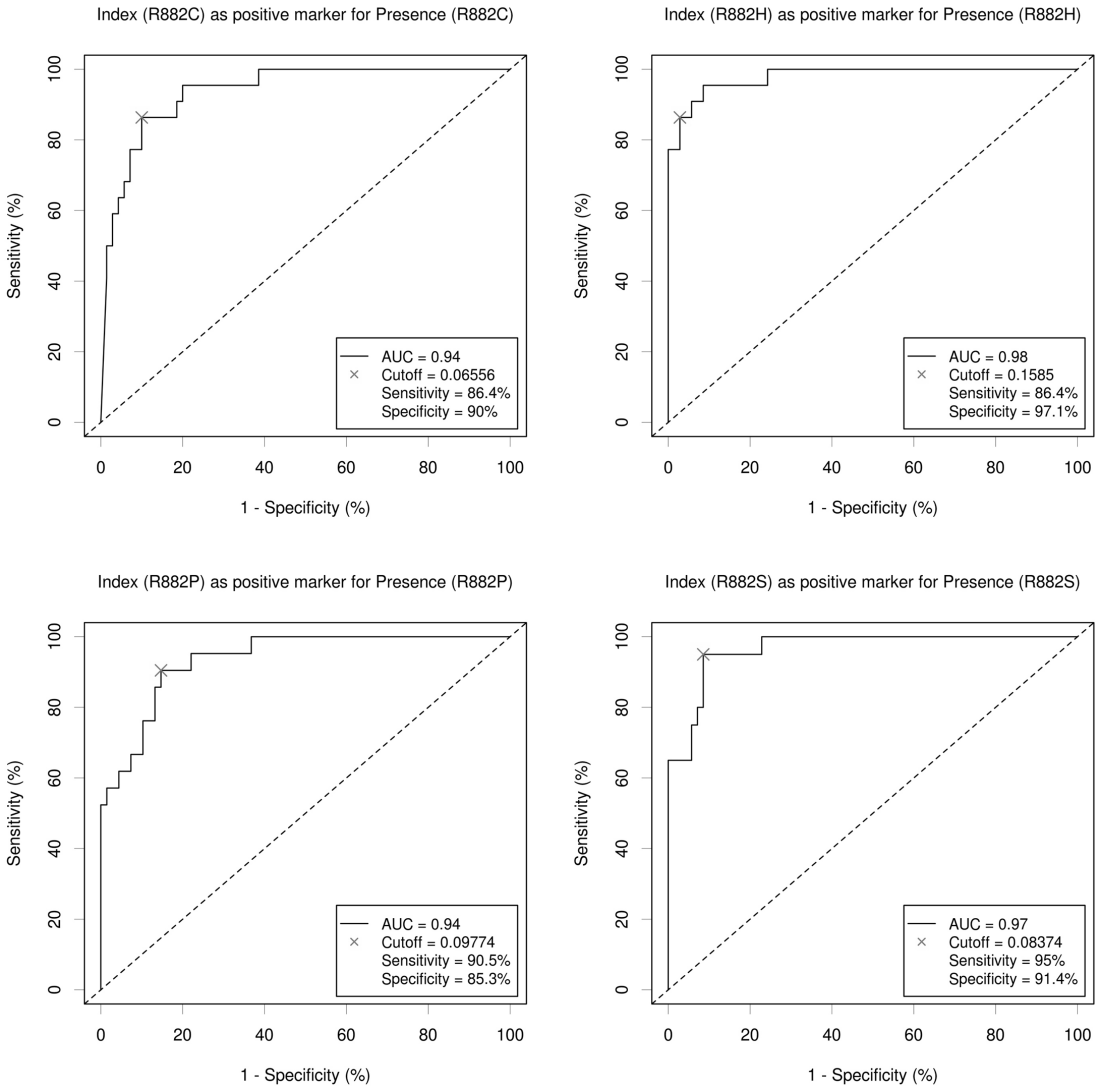
ROC curves for the determination of the overall performance of the assay and the optimal cut-off value for each mutant's index.

**Figure 3:**
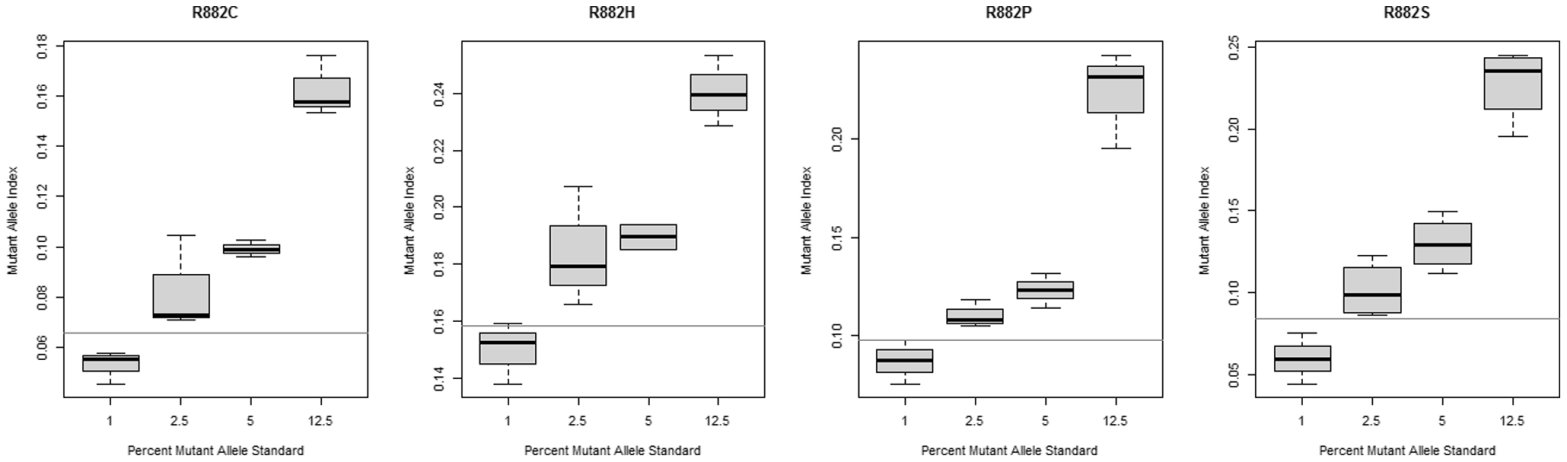
Determination of the sensitivity of the assay as expressed in percent mutant allele. The horizontal lines denote the threshold level for each mutant's index.

To determine the mutant allele index threshold values we used Receiver Operating Characteristic (ROC) analysis. ROC curves were based on the analysis of the index values from plasmid samples with wild type only (true negative samples) and wild type and mutant alleles samples (true positive samples) as described above. ROC curves for all mutants are shown at Fig. 2. The determined areas under the curve (AUC) were indicative for an excellent performance of the assay with all values being over 0.9 as follows: p.R882C (0.94), p.R882H (0.98), p. R882P (0.94), and p. R882S (0.97). The corresponding specificity and sensitivity at the selected cut-off index values are shown in Fig. 2. In order to determine the corresponding sensitivity of the assay for each mutant in terms of mutant allele burden we assayed plasmid standards with 1%, 2.5%, 5%, and 12.5% of each mutant plasmid on wild type background. The sensitivity was defined as the lowest mutant allele burden whose range of index values was above the determined respective threshold. As shown in Fig. 3 the determined sensitivity was 2.5% for all *DNMT3A* mutants.

### Concordance of the bead-based assay with direct sequencing using genomic DNA

**Figure 4:**
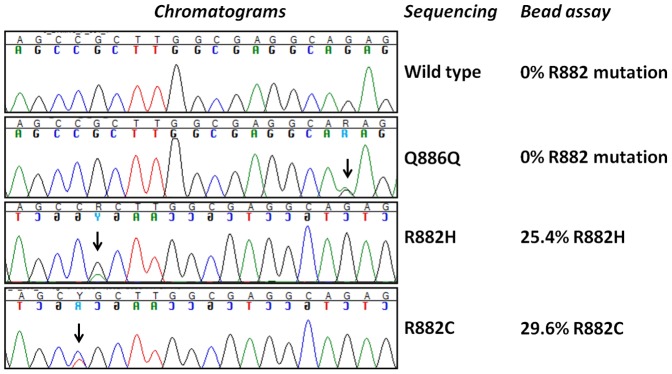
Comparison of the bead-based assay to direct sequencing in selected genomic DNA samples. The vertical arrows show the positions of the identified mutations.

We further tested the performance of our bead-based assay on a set of genomic DNA samples from 120 patients with known or suspected myeloid malignancies. We identified a total of two R882 missense mutations (one p.R882C and one p.R882Q) (Fig. 4). Both cases were AML. The results from the bead-based assay for all cases were compared with direct sequencing of exon 23 of *DNMT3A*. The sequencing data confirmed the presence of the two R882 mutations but also identified another unreported heterozygous synonymous mutation p.Q886Q (c.2658G>A). This patient was also diagnosed with AML. All other cases were negative for R882 mutations on both the microsphere-based assay and direct sequencing. This straightforward comparison validated the applicability of our assay for the detection of R882 mutations in clinical settings.

## Discussion

Recent advances in genomic profiling technologies showed that genes encoding enzymes involved in epigenetic regulation are frequently affected by the mutational process in myeloid malignancies [27]. *DNMT3A* is a key gene for the epigenetic regulation of human cells as the encoded enzyme is known to be a major player in the de novo DNA methylation at CpG sites [28]. *DNMT3A* mutational status has recently been included in several studies on the prognostic power of integrated molecular profiling in AML [9], [10], [29]. Most of the studies used targeted sequencing of exon 23 of the gene spanning the mutational hotspot, codon R882. From a clinical standpoint the restriction of molecular testing for *DNMT3A* mutations only to the mutational hotspot, codon R882, is justified by three lines of evidence: i) approximately 2/3 of all reported *DNMT3A* mutations are affecting codon R882 (with p.R882H and p.R882C being the most frequent ones); ii) R882 missense mutations perturb the normal molecular function of the enzyme; and iii) R882 mutations may differ in their prognostic effect from non-R882 mutations [18].

It was shown recently that the most frequent *DNMT3A* mutation, R882H, disrupted the dimerization interface of the enzyme. This leads to a several fold drop down in its activity as a processive CpG methylase [30]. However, *DNMT3A* R882 mutations are not just hypomorphic and they seem to exert also dominant negative effect as the expression of the analogous mouse *DNMT3A* R878H mutations in embryonic stem (ES) cells containing endogenous DNMT3A or DNMT3B caused hypomethylation [31]. Xu et al. [32] just showed that the transplantation of hematopoietic stem cells (HSCs) transduced retrovirally with R882H mutant and transplanted to immunodeficient mice caused CMML disease with characteristic hypomethylation patterns. A number of retrospective clinical studies assessed the prognostic power of *DNMT3A* in AML. Only a few of them, however, compared R882 mutations with non-R882 mutations, but the conclusions regarding their prognostic power are not consistent. For instance, Gaidzik et al. [7] showed that R882-mutated cases may be prognostic for unfavorable OS vs. non-R882-mutated cases. Other studies, however, did not find such a difference between different types of DNMT3A mutations [33]. Taken together, the clinical and experimental evidence suggest the relevance of *DNMT3A* mutations detection in the work-up for AML (and eventually other related myeloid malignancies).

To address the rapidly growing need for testing the *DNMT3A* R882 in clinical settings we adapted the bead-based flow platform developed by the Luminex Corporation, USA. This system utilizes barcoding of various analytes recognizing molecules (antibodies, ligands, oligonucleotides) through coupling with fluorescently labeled microbeads [34]. In the cases of direct hybridization based method the amplified DNA fragment is either directly labeled with Cy3/PE or with biotin with subsequent streptavidin-phycoerythrine (SAPE) labeling. A number of recent reports demonstrated the applicability of this technique for detection and/or quantitation of somatic mutations associated with malignant diseases such as *KRAS* and *BRAF* [35], [36], *NPM1*, *JAK2*, *MPL, IDH1/2* [19], [20], [21], [37], [38], [39] and for detection of chromosomal translocations [40], [41] and even for CpG methylation profiling [42]. The greatest advantage of this method is the opportunity for multiplexing in a single tube the detection of up to 100 analytes. A nice demonstration of that advantage for detection of cancer-associated mutations has recently been provided by Bando et al. [43] who performed multiplex single tube testing for 36 mutations in *KRAS*, *NRAS*, *BRAF* and *PIK3CA*. Unfortunately, they did not provide any data on the analytical sensitivity of the assay. The greatest challenge in the development of such assays is the design of probes that could specifically discriminate between sequences differing in just one nucleotide base. In the last decade a working strategy to overcome this limitation appeared to be the use of synthetic nucleotides that can increase the specificity and the Tm of the oligonucleotide probes they are incorporated in. The LNA-containing oligonucleotides have been reported to be successfully coupled with Luminex beads for SNP or somatic mutations detection. Here, to the best of our knowledge we reported for the first time the use of BNA(NC)-containing oligonucleotides coupled with micro-beads and resolved on the Luminex platform. Our in-house designed BNA(NC) probe set appeared to be superior to an LNA probe set intended for the same purpose. The BNA(NC) incorporation allowed for more uniform Tm of the probes in the set resulting in better sensitivity and specificity as demonstrated by the very high linearity between the mutant allele standards and the obtained mutant allele indices (Fig. 1 and Table 2). This BNA(NC)-modified probes superiority in comparison to the LNA-modified ones might not be considered surprising as this has already been shown for RNA silencing application [44].

We further used a state-of-the art technique to determine the index cut-off value for presence of any of the specific mutant alleles. Notably, all the cut-off values translated in 2.5% mutant allele burden sensitivity, which is of clinical relevance, as this value would mean 5% heterozygous mutant carrier cells in the sample. In principle enhancement of the sensitivity of the assay is possible through use of mutant-enrichment PCR protocols (COLD-PCR) or signal amplification techniques (e.g. use of PE-labeled anti-biotin antibodies). This would, however, increase the work load associated with the assay. We also validated our multiplex assay on clinical samples from patients with known or suspected myeloid malignancies. We were able to identify two R882 mutations, both in AML patients. This result was concordant with the direct sequencing read-out which also confirmed the presence of these mutations. Expectedly, sequencing appeared to be superior in detecting other mutations beyond the R882 codon. We identified a synonymous p.Q886Q mutation (c.2658G>A), which is obviously of no clinical relevance.

In conclusion, here we demonstrated the applicability of BNA(NC) probes coupled with fluorescently labeled beads for quantitative detection of DNMT3A R882 mutations. This method is rapid (taking up to 5 hours from DNA extraction to data acquisition) and allows processing of samples in a mid-throughput format (in 96 or 384 well plates). The demonstrated sensitivity of 2.5% for each mutant allele is well suited for the clinical practice. The greatest advantage of this assay is the opportunity for further multiplexing of the assay for simultaneous detection of many clinically relevant mutations. Therefore, it may become a valuable tool in the era of integrated integrated mutational profiling of myeloid malignancies especially for laboratories not having access to NGS platforms.
